# The Efficacy of Platinum Chemotherapy in a Japanese Malignant Melanoma Patient With a BRCA2 Mutation Identified by Gene Panel Testing

**DOI:** 10.7759/cureus.45572

**Published:** 2023-09-19

**Authors:** Reiko Takayanagi-Hara, Natsuko Saito-Sasaki, Etsuko Okada, Yu Sawada

**Affiliations:** 1 Dermatology, University of Occupational and Environmental Health, Kitakyushu, JPN

**Keywords:** case study, without chemotherapy, platinum based, malignant melanoma metastasis, brca2 mutation

## Abstract

A BRCA2 mutation increases the chance of developing cancer and has been linked to several diseases, including hereditary breast, ovarian, pancreatic, and prostate cancers. We present a case of advanced malignant melanoma treated with platinum-containing chemotherapy and demonstrate a momentarily favorable clinical outcome as determined by a Next Generation Sequencer (NGS) gene panel testing. A 54-year-old female with BRAF wild-type of anal primary melanoma received adjuvant immunotherapy with nivolumab following surgical resection. Novel distant lung metastasis was identified four months after the adjuvant therapy. Multi-gene panel testing figured out another potential treatment strategy using a sample from a distant metastatic tumor and identified a BRCA2 mutation in the tumor. Based on the sensitivity to platinum agents in BRCA2 mutation-positive tumors, DAC-Tam therapy (Dacarbazine, Nimustine, Cisplatin, and Tamoxifen) was administrated and showed tumor size reduction. After five rounds of DAC-Tam treatment, the metastatic lesion decreased from 17 mm to 5 mm. The parent was treated with platinum and Dacarbazine alone because of deteriorated renal function and grade 3 myelosuppression. In addition, the tumor showed resistance to the platinum plus Dacarbazine chemotherapy. Her chemotherapy-induced renal failure and bone marrow suppression did not improve well. Additionally, she felt significant weakness due to poor dietary intake and did not want to receive additional chemotherapy. To relieve her symptoms, she and her family desired the best supporting care and moved her to another hospital. The patient died 12 months after submitting the gene panel.

## Introduction

A BRCA2 mutation increases the chance of developing cancer and has been linked to several diseases, including hereditary breast, ovarian, pancreatic, and prostate cancers. With the current development of the Next Generation Sequencer (NGS), it has been determined that BRCA2 mutations are also present in individuals with melanoma. The specific definition of the Asian population is yet unknown. Here, we present a case of advanced malignant melanoma treated with platinum-containing chemotherapy and demonstrate a momentarily favorable clinical outcome as determined by an NGS gene panel testing.

## Case presentation

A 54-year-old female with BRAF wild-type of anal primary melanoma received adjuvant immunotherapy with nivolumab following surgical resection. Distant lung metastasis was identified four months after the adjuvant therapy. Therefore, the therapy was changed to nivolumab plus ipilimumab combination therapy; unfortunately, this treatment was also ineffective in regulating distant metastasis. To identify another therapeutic candidate option, we performed multi-gene panel testing to figure out another potential treatment strategy using a histological sample from a distant metastatic tumor and identified a BRCA2 mutation in the tumor. Due to prior research demonstrating the effectiveness of platinum-based chemotherapy in treating cancers with the BRCA2 mutation, DAC-Tam therapy (Dacarbazine, Nimustine, Cisplatin, and Tamoxifen) was administrated. After five rounds of DAC-Tam treatment, the metastatic lesion decreased from 17 mm to 5 mm. Six months after the start of the therapy, metastatic tumors reduced tumor size. The patient was treated with platinum and Dacarbazine alone after that because renal function deteriorated and there was grade 3 myelosuppression. Especially, at the beginning of the DAC-Tam treatment, renal function was maintained at 108 mL/min/1.73 m^2^; however, it fell to 38 mL/min/1.73 m^2^ after five rounds of chemotherapy. Her chemotherapy-induced renal failure and bone marrow suppression did not improve well. Additionally, she felt significant weakness due to poor dietary intake and did not want to receive additional chemotherapy. Furthermore, the tumor became resistant to the platinum plus Dacarbazine chemotherapy. To relieve her symptoms, she and her family desired the best supporting care and moved her to another hospital. The patient died 12 months after submitting the gene panel.

## Discussion

The tumor suppressor genes BRCA1 (on chromosome 17) and BRCA2 (on chromosome 13) maintain chromosomal integrity and are essential for the cellular response to double-stranded DNA breaks [1]. While BRCA1 mutations have not been found to be significantly associated in large retrospective familial studies [1], BRCA2 mutations in melanoma have been found in 2.3% of melanoma cases in the North American population [2], and the BRCA2 variant was significantly more prevalent in melanoma patients than in control subjects in the Polish population (odds ratio: 1.8) [3], suggesting that the frequency of BRCA2 mutations may be influenced by regional factors. Malignancies with the BRCA2 mutation demonstrate the effectiveness of platinum chemotherapy as a benefit of the diagnosis of this mutation [4]. Since our case demonstrated ephemeral effectiveness and eventually resistance to platinum-based chemotherapy, more alternative treatment strategies would be sought to enhance the therapeutic result even in the case of a BRCA2 mutant melanoma.

Serious side effects of Cisplatin, including renal dysfunction, myelosuppression, hearing impairment, visual impairment, and interstitial pneumonia, have been reported [5]. Therefore, we conducted adequate rehydration to avoid these adverse events. However, adverse events, especially renal dysfunction, could not be prevented.

Since the detailed effectiveness of Cisplatin in treating BRCA2 mutant melanomas has not been reported in the Asian population, further case series studies are required to clarify the actual influence of this chemotherapy.

## Conclusions

Our case illustrated a novel therapeutic approach for inoperable melanoma by utilizing the current therapy options. By detecting the precise gene mutation, gene panel testing may lead to a breakthrough in the treatment of advanced, incurable malignant melanoma. The effectiveness of these medications in treating BRCA2 mutant melanomas in Asian populations has to be clarified; hence, further case studies are required in this area.
